# Clinical and laboratory features associated with serum phosphate concentrations in malaria and other febrile illnesses

**DOI:** 10.1186/s12936-020-03166-z

**Published:** 2020-02-21

**Authors:** Ho-Ming E. Suen, Geoffrey Pasvol, Aubrey J. Cunnington

**Affiliations:** 1 Faculty of Medicine, Imperial College London, London, UK; 2 Department of Life Sciences, Imperial College London, London, UK; 3 Section of Paediatric Infectious Disease, Department of Infectious Disease, Imperial College London, London, UK

**Keywords:** Phosphate, Temperature, Platelets, Infection, Malaria, Hypophosphatemia

## Abstract

**Background:**

Hypophosphatemia is common in severe infections including malaria. Previous studies suggested that serum phosphate concentrations correlate with temperature, but it is unclear whether the type of infection and other factors occurring during infection influence this association. Here relationships were investigated between serum phosphate levels, cause of fever, demographic, clinical and laboratory parameters.

**Methods:**

Anonymized data were analysed from 633 adults with malaria or other febrile illness admitted to Northwick Park Hospital, London, UK. Univariable and multivariable generalized linear model analyses were performed to examine associations with serum phosphate levels. Interaction terms were included to investigate whether cause of fever (malaria vs other illness), malaria parasite species, or malaria severity influenced the association of other variables with phosphate.

**Results:**

Hypophosphatemia was common in subjects with malaria (211/542 (39%)), and in other febrile illnesses (24/91 (26%)), however median phosphate levels did not differ significantly by diagnostic group, parasite species or severity of malaria. In all analyses, there were highly significant negative associations between serum phosphate and axillary temperature, and positive associations between serum phosphate and platelet count. There were no significant interactions between these variables and cause of fever, parasite species or severity of illness. Sodium and potassium concentrations were associated with serum phosphate in subjects with malaria and when data from all subjects was combined.

**Conclusion:**

Serum phosphate is consistently associated with temperature and platelet count in adults with diverse causes of fever. This may be a consequence of phosphate shifts from plasma into cells to support ATP generation for thermogenesis and platelet activation.

## Background

Abnormal serum phosphate levels are a common finding in seriously ill patients, and hypophosphatemia (usually defined as serum phosphate < 0.8 mmol/L) is commonly seen in patients with severe infections [1–9]. Associations between hypophosphatemia and fever [10] or hyperthermia [11] have been previously reported, and some studies have suggested that hypophosphatemia may be a useful predictor of mortality [2, 3]. Hypophosphatemia is well described in patients with malaria [7, 8, 10, 12], and a strong correlation between body temperature and serum phosphate concentration was seen in one study of patients with *Plasmodium vivax* malaria [10].

Serum phosphate concentration is usually maintained in the range 0.8–1.5 mmol/L in adults [9, 13]. Phosphate is essential to the biology of life, as the fundamental energy currency of cells in adenosine triphosphate (ATP), as a critical component of nucleic acids, as a molecular switch controlling protein function, as a component of membrane phospholipids, and as a buffer to changes in pH [13]. Most phosphorous in the human body is stored in bones and teeth, and the rest is predominantly intracellular, where concentrations are 100 times greater than in plasma [9, 13]. Only about 1% of body phosphorous content is contained in the plasma, but this phosphate is important because it is the medium of exchange between different body compartments, particularly when phosphate is required rapidly [9, 13, 14]. Phosphate homeostasis is maintained by the action of parathyroid hormone (PTH), calcitriol, and increasingly well-characterized phosphatonin peptides, which regulate uptake from the intestine, reabsorption by the kidney, and phosphate exchange between bone and extracellular pools [9, 13–15]. Phosphate is co-transported with sodium by multiple tissue-specific phosphate transporters, and this transport is influenced by potassium and acid–base status [15].

Mild hypophosphatemia is often asymptomatic, but with more severe hypophosphatemia symptoms such as weakness, malaise, anorexia, and bone pain, develop, and in extreme cases haemolysis, rhabdomyolysis, coma, and death have been reported [9, 16]. Many of these features are seen in patients with malaria and sepsis.

Hypophosphatemia may be caused by reduced intake or absorption from the gut, increased loss of phosphate (usually from the kidney), or redistribution through transcellular shifts [9]. The contributions of these mechanisms to hypophosphatemia in severe infection are not well elucidated. Proposed explanations include increases in glycolysis [10, 17], urinary excretion of phosphate [7], and respiratory alkalosis associated with increased respiratory rate and depth [17]. High levels of pyrogenic proinflammatory cytokines, such as tumour necrosis factor (TNF) and interleukin-6 (IL-6) have been correlated with hypophosphatemia, and can induce hypophosphatemia when administered experimentally [4].

In the present study, the associations between serum phosphate concentrations and temperature, other clinical variables, and other laboratory variables, were investigated in a large cohort of subjects with malaria and a comparison group of subjects with other causes of febrile illness. It was hypothesized that temperature would influence phosphate concentrations, as observed in a previous study [10], but that the cause of fever (malaria vs other febrile illness, or *Plasmodium falciparum* vs non-*P. falciparum* malaria) would also influence phosphate concentrations, and that lower phosphate concentrations would be associated with more severe disease.

## Methods

### Data collection

Anonymized data were extracted from an audit database of febrile patients admitted to the infectious disease ward of Northwick Park Hospital from April 1991 through to May 2006 (the malaria dataset, which has been previously been reported [18]) and from January 2009 through to March 2010 (non-malarial febrile illnesses). Patients in the malaria group were infected with either *P. falciparum*, *P. vivax*, *Plasmodium ovale* or *Plasmodium malariae* and included both severe and uncomplicated infections. Severe malaria was defined as reported previously using World Health Organization (WHO) criteria [18]. The other febrile illness group consisted of patients without malaria who had been admitted with a broad range of febrile diseases (including suspected and proven bacterial, viral, parasitic and tuberculosis infections, see Additional file 1). Clinical observations such as axillary temperature and laboratory blood results were recorded at the time of the blood draw for serum phosphate measurement (see Additional file 2). All blood biochemistry and haematology results were obtained from the corresponding accredited clinical laboratories at Northwick Park Hospital, London, UK. The normal range for serum phosphate concentrations defined by this laboratory was 0.75–1.3 mmol/L; therefore values below 0.75 mmol/L were defined as hypophosphatemia, and values above 1.3 mmol/L were defined as hyperphosphatemia for the purpose of this study.

### Exclusions

Patients with a clinician diagnosis of renal failure, or blood creatinine level ≥ 265 μmol/L [19] were excluded from the study, given the established association of renal failure with hyperphosphatemia [9]. In total, 12 patients were excluded (11 with malaria and 1 with other febrile illness). The median serum creatinine and phosphate levels for the excluded subjects were 348 μmol/L and 1.39 mmol/L, respectively.

### Data analysis

All statistical analyses used two-sided tests and *P *< 0.05 was considered to be significant. Categorical and continuous data were compared between groups using the Chi squared and unpaired Mann–Whitney tests, respectively. Four variables (age, creatinine, white blood cell count, platelet count) were log transformed before regression analyses in order to normalize their distributions. Univariable and multivariable generalized linear model (glm) analyses were performed using the R statistical software. Correlation plots were generated with the ggplot2 package and regression lines with 95% confidence intervals were constructed with the linear model method. Variables with significant associations (*P *< 0.05) with phosphate in univariable analysis were included in multivariable models. Forward and backward selection were used to determine the optimal multivariable model in which the Akaike information criterion was minimized whilst all variables were significantly (*P *< 0.05) associated with phosphate.

## Results

633 subjects were included in the study, 542 with malaria and 91 with other febrile illnesses (Table 1). Age and gender distributions were similar between groups, but there was a significant difference in ethnicity, with a greater proportion of malaria subjects being of African origin, and a greater proportion of Asian and Caucasian subjects in the other febrile illness group. Temperature and creatinine levels were significantly higher in the malaria group than in the other febrile illness group, while platelet count, white cell count, corrected calcium, and potassium were all lower in the malaria group than in the other febrile illness group. Median levels of serum phosphate did not differ between the two groups. However, when serum phosphate concentrations were categorized as either low, normal or high, the distribution of phosphate concentrations in subjects with malaria was revealed to be different to that in the other febrile illness group. Subjects with malaria were significantly more likely to have both hypo- (211/542 (39%) vs 24/91 (26%)) and hyperphosphatemia (59/542 (11%) vs 4/91 (4%)) and less likely to have normal phosphate levels (272/542 (50%) vs 63/91 (69%)) (Chi squared test *P *= 0.002).

**Table 1:** Summary of demographic, clinical and laboratory parameters in subjects with malaria and subjects with other febrile illness

	Malaria (n = 542)	Other febrile illness (n = 91)	*P*
Sex	M: 353 (65%), F: 189 (35%)	M: 53 (58%), F: 38 (42%)	0.205
Ethnicity	AS: 191 (35%), AF: 273 (50%), C: 78 (14%)	AS: 53 (59%), AF: 4 (4%), C: 33 (37%)^a^	< 0.001

	Median [IQR]	Median [IQR]	
Age	35 [26–47]	35 [28–48]	0.491
Temperature (°C)	38.0 [37.2–39.0]	37.7 [36.3–40.0]	0.026
Hemoglobin (G/Dl)	12.9 [11.4–14.3]^b^	13.3 [12.1–14.5]	0.106
Wbc (× 10^9^/L)	5.4 [4.4–6.6]	9.6 [6.4–13.1]	< 0.001
Platelet (× 10^9^/L)	102 [65–153]^c^	253 [192–334]	< 0.001
Corrected calcium (Mmol/L)	2.23 [2.16–2.30]^d^	2.37 [2.32–2.44]	< 0.001
Phosphate (Mmol/L)	0.88 [0.58–1.12]	0.89 [0.73–1.02]	0.729
Sodium (Mmol/L)	137 [134–139]	136 [133–138]^a^	0.068
Potassium (Mmol/L)	3.9 [3.6–4.2]	4.1 [3.9–4.4]	< 0.001
Creatinine (Mmol/L)	96 [82–110]	76 [67–87]	< 0.001

*m* male, *f* female, *AS* Asian, *AF* African, *C* Caucasian, *WBC* White Blood Cell count, *IQR* interquartile range

^a^n = 90; ^b^n = 541; ^c^n = 540; ^d^n = 539. *P* for two-sided Chi squared tests for categorical variables and for Mann–Whitney test for continuous variables

Amongst the subjects with malaria, 404 (75%) had *P. falciparum* infection and 138 (25%) non-*P. falciparum* infections (*P. vivax* (n = 120), *P. ovale* (n = 10), and *P. malariae* (n = 8)). 60 (11%) of the malaria infections (all *P. falciparum*) were severe. There were no significant differences between the two groups in terms of gender or age. As expected, the proportion of African patients was greater in the *P. falciparum* group and the proportion of Asian patients was greater the non-*P. falciparum* group (Table 2). Creatinine levels and hemoglobin were significantly higher, and white cell count was significantly lower in the *P. falciparum* group than in the non-*P. falciparum* group. Median levels of serum phosphate were similar between the two groups (Table 2), but abnormal phosphate levels were slightly less common in *P. falciparum* than non-*P. falciparum* infections (hypophosphatemia in 154/404 (38%) vs 57/138 (41%), respectively; hyperphosphatemia in 37/404 (9%) vs 22/138 (16%), respectively; normal phosphate in 213/404 (53%) vs 59/138 (43%), respectively; Chi squared test *P *= 0.036).

**Table 2:** Summary table of demographic, clinical and laboratory parameters in subjects with *P. falciparum* and non-*P. falciparum* malaria

	*P. falciparum* (n = 404)	Non-*P. falciparum* (n = 138)	*P*
Sex	M: 274 (68%), F: 130 (32%)	M: 79 (57%), F: 59 (43%)	0.024
Ethnicity	AS: 79 (20%), AF: 257 (64%), C: 68 (17%)	AS: 112 (81%), AF: 16 (12%), C: 10 (7%)	< 0.001
Severe	60 (15%)	0 (0%)	< 0.001

	Median [IQR]	Median [IQR]	
Age	35 [27–46]	36 [25–50]	0.988
Temperature (°C)	38.0 [37.2–39.0]	38.0 [37.2–39.0]	0.489
Hemoglobin (g/dL)	13.1 [11.7–14.5]^a^	12.4 [10.9–13.8]	0.001
WBC (× 10^9^/L)^3^	5.3 [4.3–6.5]	5.8 [4.9–7.0]	0.006
Platelet (× 10^9^/L)	101 [65–152]^a^	103 [74–154]^b^	0.305
Corrected calcium (mmol/L)	2.23 [2.16–2.29]^a^	2.24 [2.16–2.30]^c^	0.375
Phosphate (mmol/L)	0.87 [0.60–1.12]	0.93 [0.52–1.14]	0.851
Sodium (mmol/L)	136 [134–139]	137 [135–140]	0.063
Potassium (mmol/L)	3.9 [3.6–4.2]	3.9 [3.7–4.1]	0.889
Creatinine (μmol/L)	98 [84–112]	90 [70–104]	< 0.001

*M* male, *F* female, *AS* Asian, *AF* African, *C* Caucasian, *WBC* White blood cell count

^a^n = 403; ^b^n = 137; ^c^n = 136. *P* for two-sided Chi squared tests for categorical variables and for Mann–Whitney test for continuous variables

**Fig. 1:**
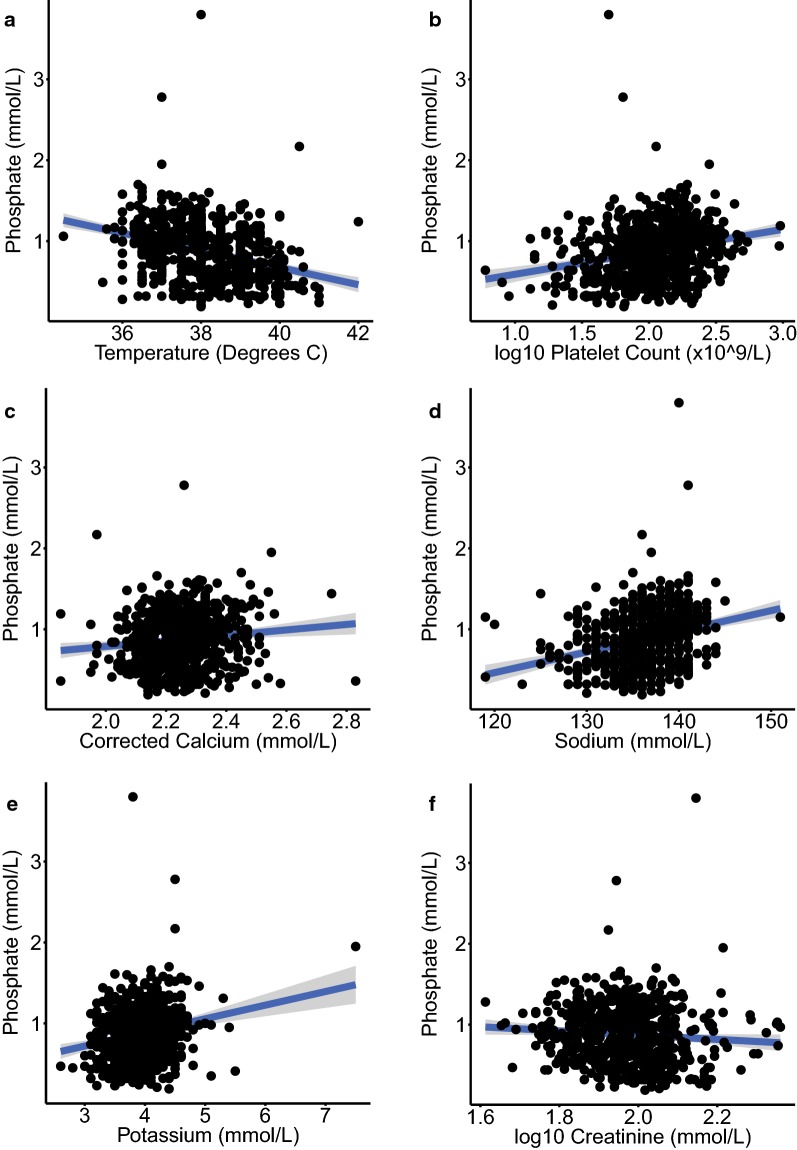
Correlation between serum phosphate concentration and other variables. Plots **a–f** illustrate the correlation between serum phosphate and other variables which were found to be significantly associated with phosphate in univariable analyses of the combined dataset of subjects with malaria and other febrile illness: **a** temperature; **b** platelet count; **c** corrected calcium; **d** sodium; **e** potassium; **f** creatinine. Regression lines with 95% confidence intervals are shown

Associations between serum phosphate levels and demographic, clinical, and laboratory parameters were evaluated in univariable analyses using the combined dataset of 633 subjects with malaria and other febrile illnesses. Temperature, log_10_(platelet count), corrected calcium, sodium, potassium and log_10_(creatinine) were all significantly associated with serum phosphate (Table 3 and Fig. 1). Multivariable analysis revealed significant independent associations between serum phosphate and temperature, log_10_(platelet count), sodium and potassium. A strong negative association was observed between temperature and serum phosphate, with a 0.082 mmol/L decrease in serum phosphate associated with each 1 °C rise in temperature in the multivariable model. Significant positive associations were observed between serum phosphate and log_10_(platelet count), sodium and potassium. Although malaria was not associated with phosphate concentration in the univariable analyses, it was considered that malaria status may influence the relationships between phosphate and temperature or platelets. However, there were no significant statistical interactions between malaria status and these variables in the multivariable model.

**Table 3:** Association of demographic, clinical and laboratory parameters with serum phosphate levels in the combined dataset

Variable	Univariable analysis				Multivariable analysis			
	Estimate	Std. Error	t value	*P*	Estimate	Std. Error	t value	*P*
Sex: male	− 0.028	0.03	− 0.96	0.34				
Ethnicity: Asian	− 0.019	0.031	− 0.6	0.55				
Ethnicity: Caucasian	− 0.029	0.04	− 0.71	0.48				
log_10_(age (years))	0.015	0.086	0.17	0.87				
Temperature (°C)	− 0.11	0.012	− 9.2	< 2 × 10^−16^	− 0.082	0.011	− 7.2	1.9 × 10^−12^
Hemoglobin (g/dL)	− 0.0098	0.0068	− 1.4	0.154				
log_10_(White cell count (× 10^9^/L))	− 0.016	0.078	− 0.2	0.841				
log_10_(Platelet count (× 10^9^/L))	0.28	0.044	6.3	5.4 × 10^−10^	0.15	0.043	3.4	0.00082
Corrected Calcium (mmol/L)	0.34	0.11	3	0.0032				
Sodium (mmol/L)	0.026	0.0035	7.3	1.0 × 10^−12^	0.017	0.0035	4.9	1.4 × 10^−6^
Potassium (mmol/L)	0.17	0.033	5.1	4.8 × 10^−7^	0.12	0.031	3.85	0.000129
log_10_(Creatinine mmol/L)	− 0.26	0.13	− 2	0.043				
Malaria: yes	− 0.004	0.04	− 0.099	0.921				

Variables with a significant association (*P *< 0.05) in the univariable analysis entered the model selection in the multivariable analysis. Results are only shown for those variables which were significant in the final multivariable model. *Std. Error* standard error. n = 633

Next, the malaria dataset alone was analysed, to determine whether there were any associations with phosphate which were unique to malaria or associated with parasite species. Univariable analysis revealed associations between serum phosphate and temperature, log_10_(platelet count), corrected calcium, sodium, potassium and log_10_(creatinine). This was consistent with the findings in the combined dataset. Neither disease severity, nor parasite species, were significantly associated with phosphate concentration in univariable analysis (Table 4). The best multivariable model in the malaria dataset included the same variables as those selected in the combined dataset, and consisted of temperature, log_10_(platelet count), sodium and potassium. A strong negative association was again observed between temperature and serum phosphate, while significant positive associations were observed with platelet count, sodium and potassium (Table 4). There were no significant interactions between parasite species or malaria severity and temperature or platelets in the multivariable model. The analyses above suggested that the larger malaria dataset probably dominated any associations with phosphate seen in the combined dataset analysis. Therefore, the same analyses were repeated in the group of 91 subjects with other febrile illness alone. Univariable analysis indicated significant associations between serum phosphate and temperature, log_10_(platelet count), corrected calcium, and potassium. In multivariable analysis, the best model included only temperature and log_10_(platelet count). Serum phosphate was negatively associated with temperature and positively associated with log_10_(platelet count) (Table 5).

**Table 4:** Association of demographic, clinical and laboratory parameters with serum phosphate levels in the malaria dataset

Variable	Univariable analysis				Multivariable analysis			
	Estimate	Std. Error	t value	*P*	Estimate	Std. Error	t value	*P*
Sex: male	− 0.024	0.034	− 0.73	0.47				
Ethnicity: Asian	− 0.029	0.035	− 0.81	0.42				
Ethnicity: Caucasian	− 0.013	0.048	− 0.28	0.78				
log_10_(Age (years))	0.0045	0.098	0.046	0.96				
Temperature (°C)	− 0.11	0.012	− 8.6	< 2 × 10^−16^	− 0.086	0.013	− 6.8	2.2 × 10^−11^
Hemoglobin (g/dL)	− 0.011	0.0076	− 1.5	0.14				
log_10_(White cell count (× 10^9^/L))	− 0.025	0.11	0.24	0.81				
log_10_(Platelet count (× 10^9^/L))	0.36	0.054	6.6	9.0 × 10^−11^	0.2	0.054	3.7	0.0002
Corrected calcium (mmol/L)	0.38	0.14	2.7	0.0068				
Sodium (mmol/L)	0.029	0.0039	7.4	6.0 × 10^−13^	0.017	0.0039	4.4	1.5 × 10^−5^
Potassium (mmol/L)	0.18	0.037	4.7	3.1 × 10^−6^	0.13	0.035	3.9	0.00012
log_10_(Creatinine mmol/L)	− 0.31	0.15	− 2.1	0.037				
Species: Non- *P. falciparum*	0.042	0.037	1.2	0.25				
Severity: severe	− 0.094	0.051	− 1.85	0.065				

Variables with a significant association (*P *< 0.05) in the univariable analysis entered the model selection in the multivariable analysis. Results of are only shown for those variables which were significant in the final multivariable model. Std. Error, standard error. n = 542

**Table 5:** Association of demographic, clinical and laboratory parameters with serum phosphate levels in the other febrile illness dataset

Variable	Univariable analysis				Multivariable analysis			
	Estimate	Std. Error	t value	*P*	Estimate	Std. Error	t value	*P*
Sex: male	− 0.05	0.052	− 0.97	0.34				
Ethnicity: Asian	− 0.032	0.13	− 0.25	0.8				
Ethnicity: Caucasian	− 0.11	0.13	− 0.88	0.38				
log_10_(age (years))	0.07	0.15	0.46	0.64				
Temperature (°C)	− 0.081	0.023	− 3.5	0.00069	− 0.071	0.023	− 3.1	0.0025
Hemoglobin (g/dL)	0.00055	0.014	0.04	0.97				
log_10_(White cell count (× 10^9^/L))	− 0.17	0.12	− 1.4	0.16				
log_10_(Platelet count (× 10^9^/L))	0.38	0.13	3	0.0037	0.31	0.12	2.5	0.013
Corrected calcium (mmol/L)	0.54	0.26	2	0.045				
Sodium (mmol/L)	0.03	0.0075	0.4	0.69				
Potassium (mmol/L)	0.15	0.066	2.3	0.024				
log_10_(Creatinine mmol/L)	− 0.059	0.28	− 0.21	0.83				

Variables with a significant association (*P *< 0.05) in the univariable analysis entered the model selection in the multivariable analysis. Results of are only shown for those variables which were significant in the final multivariable model. Std. Error, standard error. n = 91

## Discussion

The causes and effects of hypophosphatemia during infection are not fully understood, although it is plausible that severe hypophosphatemia contributes to both symptoms and adverse outcomes. Previous work has suggested that hypophosphatemia is strongly related to fever, and that there is a strong correlation between body temperature and serum phosphate in subjects with *P. vivax* malaria [10]. This study investigated whether cause of infection, body temperature, and other clinical and laboratory variables were associated with serum phosphate concentrations. To do this, patients with malaria and a heterogeneous group of patients with other causes of febrile illness were analysed. Phosphate levels were also compared between subjects with *P. falciparum* malaria and non-*P. falciparum* malaria. Multivariable models were used to identify variables with the strongest independent associations with serum phosphate levels.

Median levels of serum phosphate in the subjects in this study did not differ between those with malaria and those with other febrile illnesses, however there were differences in the distribution of phosphate levels, with malaria subjects more likely to have either hypo- or hyperphosphatemia. Similarly, there were no significant differences in median phosphate levels between *P. falciparum* and non-*P. falciparum* malaria, but there were small differences in distribution. Consistent with a previous study [10] a strong negative association between temperature and serum phosphate levels was found, but this relationship was not influenced by the cause of fever. A significant positive association between platelet count and serum phosphate levels was also found, which was not influenced by the cause of fever. Both of these associations were consistent across analysis of all subgroups. In the larger group of subjects with malaria both sodium and potassium concentrations were positively associated with phosphate concentration. These associations were not apparent when the smaller group of subjects with other causes of febrile illness were analysed alone, possibly due to the reduced power of this analysis.

This observational study does not establish whether the observed associations are the causes or effects of changes in serum phosphate, or indirectly mediated by a common unmeasured factor with a direct relationship with phosphate levels. However existing literature indicates plausible mechanisms relating all four variables identified in these multivariable models to serum phosphate concentrations. Both sodium and potassium are known to influence phosphate transport [15], and so it is unsurprising that their concentrations were associated with serum phosphate concentrations in the analyses which included the largest numbers of subjects.

The consistent association between temperature and phosphate across subgroups, and without evidence of any influence of the cause of fever or disease severity, suggests that this relationship is direct. Either temperature may increase cellular phosphate uptake or renal excretion, or phosphate transport into cells may play a causal role in changes in body temperature. Experiments in anesthetized dogs under neuromuscular blockade indicated that exogenously increasing body temperature alone did not cause a decrease in serum phosphate [11], making the former mechanisms unlikely. In contrast, increased phosphate uptake into cells may be necessary to allow an increase in energy metabolism and heat generation contributing to elevation of body temperature and fever [10, 20]. Therefore, the decrease in serum phosphate may indicate that intracellular shift of phosphate is important for the generation of fever.

An association between platelet count and serum phosphate levels has been previously reported [1]. During malaria and sepsis, decreases in circulating platelet count are mostly due to platelet activation and an associated prothrombotic state [21, 22]. Platelet activation, adhesion and aggregation are all energy-consuming processes that require a substantial amount of ATP and therefore phosphate uptake from plasma [23–26]. Thus, platelet activation may directly contribute to a decrease in serum phosphate concentrations. Additionally, platelet dense granules contain high concentrations of inorganic polyphosphate—chains of up to 100 phosphate molecules—which play a role in triggering coagulation and slowing fibrinolysis [27]. If thrombocytopenia due to platelet activation and consumption is accompanied by a compensatory increase in thrombopoiesis [28], phosphate uptake may be needed to synthesize and sequester phosphate in these polyphosphate molecules.

This study has several limitations. Phosphate levels, temperature and other variables were only analysed at a single point in time (at hospital admission), so the sequence of events cannot be determined, most importantly whether changes in phosphate concentration preceded or followed changes in temperature, platelet count and other variables. Baseline values of phosphate and other variables in each individual before they became unwell were not known, and the change from baseline may be more important than absolute values. Urinary phosphate levels, acid–base status, blood lactate, and inflammatory cytokines, were not measured, but might have been helpful to support hypotheses about mechanisms of causality. There were significant differences in ethnicity between subjects with malaria and those with non-malarial febrile illness, which might explain some of the differences in phosphate levels and associations with other variables which were observed between these groups. Within the heterogeneous group of patients with non-malarial febrile illness, further subdivision by etiology was not performed because resulting groups would be too small to undertake additional meaningful analysis. Despite these limitations, the study did have the strength of complete data on a large number of subjects for the variables of most interest.

The clinical consequences of infection-induced changes in serum phosphate are uncertain. Whilst hypophosphatemia can cause a variety of clinical features which are frequently seen in individuals with severe infections, such as weakness, malaise and alterations in mental status [9, 16], it is uncertain whether the decrease in serum phosphate induced by fever really contributes to these manifestations. Phosphate replacement for severely ill patients with very low serum phosphate levels is common practice in many countries, but it is unclear whether this has any clinical benefit [1, 29]. The potential benefits of phosphate replacement in malaria have not been evaluated, and phosphate levels are not routinely measured in malaria patients in the most endemic countries. Therefore, there is a need to prospectively evaluate the associations between serum phosphate levels, clinical and laboratory features of malaria (particularly those associated with severity), and patient outcomes, in malaria endemic countries. Phosphate infusion is a relatively straightforward, safe, and cheap intervention [1, 29], which might therefore be used in malaria patients if there was evidence to support a benefit on clinical outcomes.

To further understand the biological mechanisms underlying hypophosphatemia in infection, longitudinal studies in individuals at high risk of infection may be useful to establish the sequence of changes in phosphate, temperature and platelets. Addressing outstanding questions about the mechanisms relating these variables to serum phosphate may have relevance for therapeutics. If phosphate transport into cells is critical for pyrogenesis, transient inhibition might be a novel antipyretic approach. Similarly, inhibition of phosphate transport into platelets might be used to limit platelet activation.

## Conclusions

Increased body temperature and reduced platelet count are consistently associated with reductions in serum phosphate concentration in subjects with malaria and in subjects with other causes of fever. At present the cause and effect relationships, and therapeutic applications, remain uncertain.

## Supplementary information

**Additional file 1.** Diagnoses in subjects with other febrile illness.

**Additional file 2.** Dataset containing all analysed data from all study subjects.

## Data Availability

The dataset supporting the conclusions of this article is included within the article and its additional files.

## Abbreviation

ATP: Adenosine triphosphate
