# Supplementary material for: Clinical and laboratory features associated with serum phosphate concentrations in malaria and other febrile illnesses
Source: Malar J. 2020 Feb 21;19:85. doi: 10.1186/s12936-020-03166-z (PMC7035648; doi:10.1186/s12936-020-03166-z)
Supplement: Supplementary file 1 — Additional file 1. Diagnoses in subjects with other febrile illness. [file 12936_2020_3166_MOESM1_ESM.docx]

**Additional Table 1. Diagnoses in subjects with other febrile illness**

| Cause of fever (clinician diagnosis) | n |
| --- | --- |
| Tuberculosis | 11 |
| Pyelonephritis | 7 |
| Uncertain | 6 |
| Pneumonia | 5 |
| Possible tuberculosis | 5 |
| Viral illness (non-specific) | 5 |
| Cellulitis | 3 |
| Influenza | 3 |
| Lower respiratory tract infection | 3 |
| Urinary tract infection | 3 |
| Gastroenteritis | 2 |
| Paratypoid | 2 |
| Typhoid | 2 |
| Viral meningitis | 2 |
| Abdominal collection after Caesarean-section | 1 |
| Amoebic liver abscess | 1 |
| Bronchiectasis and recurrent pneumonia | 1 |
| Bursitis due to *Staphylococcus aureus* | 1 |
| Campylobacter gastroenteritis | 1 |
| Cholecystitis | 1 |
| Connective tissue disorder | 1 |
| Diarrhoea | 1 |
| Diverticulitis | 1 |
| E. Coli bacteremia and likely appendicitis | 1 |
| Erythema nodusum / vasculitis | 1 |
| Hepatitis unknown cause | 1 |
| Interstitial lung disease | 1 |
| Kikuchi's disease | 1 |
| Meningococcal menigitis | 1 |
| Mumps | 1 |
| Pneumococcal sepsis | 1 |
| Possible enteric fever | 1 |
| Salmonella bacteremia | 1 |
| Sjorgens syndrome | 1 |
| Skin infection | 1 |
| Staphylocococcus aureus bacteremia | 1 |
| T cell lymphoma with pneumonia | 1 |
| Uncertain, possible community acquired pneumonia | 1 |
| Uncertain, possible dengue fever | 1 |
| Uncertain, possible enteric fever | 1 |
| Uncertain, possible influenza | 1 |
| Uncertain, possible otitis media or rheumatoid arthritis associated | 1 |
| Uncertain, possible pneumonia | 1 |
| Uncertain, possible seroconversion to EBV/CMV infection | 1 |
| Varicella zoster | 1 |
| Viral hepatitis | 1 |
